# Altered motor network dynamics in myoclonus-dystonia

**DOI:** 10.1093/braincomms/fcag205

**Published:** 2026-07-21

**Authors:** Ramesh S Marapin, Harm J van der Horn, Bauke M de Jong, Elze R Timmers, Jelle R Dalenberg, Marina A J Tijssen

**Affiliations:** Department of Neurology, University Medical Center Groningen, University of Groningen, Groningen, RB 9700, The Netherlands; Expertise Center Movement Disorders Groningen, University Medical Center Groningen (UMCG), Groningen, RB 9700, The Netherlands; Department of Neurology, University Medical Center Groningen, University of Groningen, Groningen, RB 9700, The Netherlands; Department of Neurology, University Medical Center Groningen, University of Groningen, Groningen, RB 9700, The Netherlands; Department of Neurology, University Medical Center Groningen, University of Groningen, Groningen, RB 9700, The Netherlands; Expertise Center Movement Disorders Groningen, University Medical Center Groningen (UMCG), Groningen, RB 9700, The Netherlands; Department of Neurology, University Medical Center Groningen, University of Groningen, Groningen, RB 9700, The Netherlands; Expertise Center Movement Disorders Groningen, University Medical Center Groningen (UMCG), Groningen, RB 9700, The Netherlands; Department of Neurology, University Medical Center Groningen, University of Groningen, Groningen, RB 9700, The Netherlands; Expertise Center Movement Disorders Groningen, University Medical Center Groningen (UMCG), Groningen, RB 9700, The Netherlands

**Keywords:** myoclonus-dystonia, fMRI, functional connectivity, brain networks, brain dynamics

## Abstract

The exact mechanisms underlying myoclonus-dystonia (M-D) remain unknown, although the basal ganglia-thalamo-cortical (BGTC) and cerebello-thalamo-cortical (CTC) networks are hypothesized to be involved. We aimed to investigate the static and dynamic features of networks related to motor control during rest in M-D patients using functional magnetic resonance imaging (fMRI). Resting-state fMRI data from 19 M-D patients and 19 healthy volunteers were analysed. Symptom severity was measured using the Clinical Global Impression-Severity Scale, anxiety and depression with the Hospital Anxiety and Depression Scale and cognitive impairment with the Montreal Cognitive Assessment. Independent component analysis was used to identify brain components corresponding to the BGTC and CTC networks. Static within-network (i.e. spatial contribution of voxels to the average network signal time course) and between-network (i.e. correlation between network time courses) functional connectivity were examined. We additionally performed a dynamic functional connectivity analysis focused on recurrent connectivity patterns (i.e. brain states) using *k*-means clustering of windowed functional connectivity correlation matrices, whereby we identified three prototypical dynamic connectivity states in the BGTC and CTC circuits. The following brain state summary measures were computed: fraction of time spent in a state, dwell time, number of state transitions and number of state visits. Static analysis revealed that patients with M-D showed increased functional connectivity (FC) of the left supramarginal gyrus within a cognitive control network (corresponding to the cortical part of BGTC and CTC circuits), suggesting stronger integration of this area in this network (within-network *P*_fdr_ < 0.05). There were no significant group differences in between-network functional connectivity. Dynamic analysis revealed three dynamic connectivity states in BGTC and CTC circuits. Patients with M-D engaged less in a state characterized by high segregation of sensorimotor from basal ganglia and cerebellar domains, with high connectivity between networks within these domains. This finding may reflect reduced basal ganglia and cerebellar contributions during motor preparation in M-D. We found no relationships between fMRI findings and motor symptom severity in M-D patients. Our findings provide further evidence for disrupted brain network dynamics in BGTC and CTC circuits in M-D patients, supporting the hypothesis of compromised basal ganglia and cerebellar functioning. Particularly, the association of cerebellar and anterior parietal alterations is proposed to reflect impaired sensory prediction in feed-forward motor planning, potentially underlying irregular ‘non-prepared movements’.

## Teaser

Marapin *et al*. showed that patients with myoclonus-dystonia exhibit altered static and dynamic functional connectivity in networks of cognitive control, sensorimotor, subcortical and cerebellar domains. These findings may reflect impaired sensory prediction in feed-forward motor planning, potentially underlying irregular ‘non-prepared movements’.

## Graphical Abstract

**Graphical Abstract:**
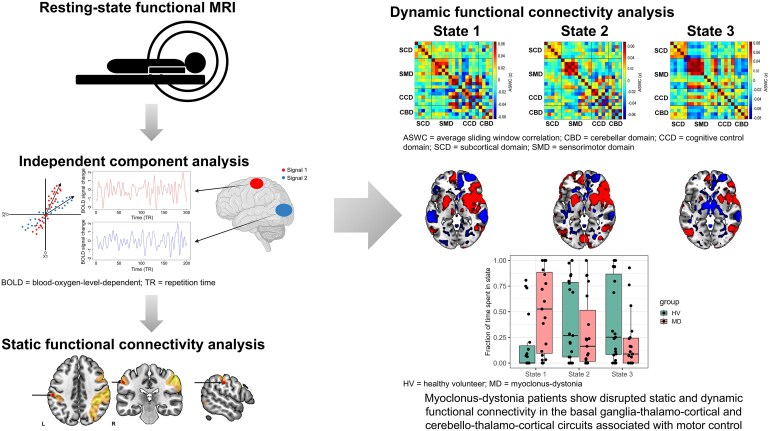

## Introduction

Myoclonus-dystonia (M-D) is a rare and genetically heterogeneous movement disorder that is clinically characterized by a combination of myoclonus and dystonia.^1^ In most cases, the predominant and most disabling motor symptom is myoclonus, presenting with very brief, ‘lightning-like’ jerks in the upper body. In addition to myoclonus, dystonia is present in most patients, usually manifesting as cervical dystonia or writer’s cramp and is often mild to moderate when present.^2^ About half of all M-D patients have a known pathogenic variant in the epsilon-sarcoglycan gene (*SGCE*), whereas in many of the remaining *SGCE*-negative carriers, the causative genes remain undetermined.^3-5^ Notably, *SGCE-*positive M-D patients have a high incidence of non-motor symptoms, especially psychiatric, compared to the general population.^1^

Currently, the exact pathophysiological mechanisms of this disorder remain unknown. For a detailed review of the literature on the pathogenesis, we refer to Roze *et al*. (2018). Over the years, a compelling argument has emerged for a cerebellar role in the pathophysiology of dystonia and myoclonus and also M-D.^6-8^ Accumulating evidence suggests that alterations in the cerebello-thalamo-cortical (CTC) network may have a prominent role in M-D, possibly linked to a GABAergic deficit indicative of Purkinje cell dysfunction. This hypothesis gains support from observations that patients with M-D exhibit significant abnormalities in a cerebellar oculomotor paradigm known as ‘saccadic adaptation’ and experience partial symptom relief from alcohol, to which the cerebellum is highly responsive.^9,10^ In line with this hypothesis of CTC network dysfunction, neuroimaging studies point to abnormalities in the brainstem, cerebellum, thalamus, insula, sensorimotor and prefrontal cortex.^11-14^ Additionally, lower membrane excitability of cortical neurons has been reported in M-D,^15^ indicating that cortical dysfunction is a contributing factor in the pathogenesis.

In addition to the CTC network, basal ganglia regions also seem to be implicated in the pathophysiology of M-D.^3^ In this regard, deep brain stimulation of the internal globus pallidus has substantial clinical benefit for treating M-D. Intriguingly, micro-recording studies performed during deep brain stimulation have shown abnormal neuronal activity in the globus pallidus of M-D patients. In dystonia, an imbalance in striatal dopaminergic function is thought to result in abnormal thalamo-motor-cortical hyperexcitability,^16^ disrupting the control of fine-tuned movements. Therefore, it seems that both the basal ganglia-thalamo-cortical (BGTC) and CTC networks are implicated in M-D, though it remains unclear how these networks functionally interact.

To address this question, resting-state functional MRI (rsfMRI) can be employed to investigate how large-scale functional brain networks interact and can provide unique insights into BGTC and CTC network functioning in M-D. Resting-state functional MRI measures spontaneous low-frequency fluctuations in the blood-oxygen-level–dependent (BOLD) signal in the brain during rest.^17^ In this context, highly synchronized brain regions correspond to functionally relevant intrinsic resting-state networks.^18^ These resting-state networks can then be further explored, for example, by investigating functional connectivity (FC) within- and between-networks, which reflect functional communication between brain regions.^19^

Importantly, most rsfMRI studies analyse brain networks by averaging connectivity over the entire duration of an fMRI scan, which implies identification of brain connectivity that remains relatively constant over time, also referred to as ‘static functional connectivity’. However, dynamic integration and orchestration of behaviour across multiple time scales make clear that resting-state connectivity is not static but has ‘dynamic’ features, which have indeed been demonstrated by fMRI studies addressing this issue.^20,21^ In a dynamic FC analysis, recurring patterns of network connectivity can be clustered into brain ‘connectivity states’, which can be considered prototypical FC patterns that subjects tend to return to across time.^22^ Previous studies investigating changes and differences in these dynamic patterns have uncovered new network abnormalities across movement disorders.^23,24^

In this exploratory study, we aimed to use rsfMRI to investigate the static and dynamic functioning of brain regions that are part of the BGTC and CTC brain networks involved in motor control in patients with M-D, as these networks are thought to play a role in this disorder. A better understanding of the pathophysiology could lead to improved treatment options for patients with M-D.

## Materials and methods

### Participants

The current study is part of the larger Next Move in Movement Disorders (NEMO) study. In NEMO, participants also underwent movement registration tasks,^25^ task-based fMRI and ^18^F-FDG PET scans. Here, we focus on resting-state fMRI analysis.

Nineteen patients with a clinical diagnosis of M-D were recruited from movement disorder clinics from the UMCG and one from the Academic Medical Center, Amsterdam, the Netherlands. The diagnosis was confirmed by a movement disorders expert (M.T.). Ten (of the 19) patients had a confirmed pathogenic variant in the *SGCE* gene. Nineteen age- and sex-matched healthy volunteers were recruited through advertisements in the UMCG or were acquaintances of researchers involved in the NEMO study.

Participants were included if they were at least 16 years of age. Exclusion criteria included the following: (i) other neurological conditions that lead to movement problems other than the hyperkinetic movement disorder, (ii) other conditions that lead to impaired hand or arm function, (iii) participants with a pacemaker and (iv) contraindications for MRI scanning. In addition to these exclusion criteria, healthy volunteers who were first-degree relatives of patients with hyperkinetic movement disorders were additionally excluded. None of the participants were taking psychoactive medications at the time of scanning that are known to affect resting-state fMRI signals. Additionally, no patients were under the effect of recently administered botulinum toxin treatment during the MRI sessions.

This study was approved by the medical ethical committee of the UMCG (METc 2018/444). Written informed consent was obtained from all subjects according to the Declaration of Helsinki.

### Clinical evaluation

Clinical characteristics and demographics of the patients were obtained during their study visit. For a complete description of the collected clinical information, we refer the reader to the NEMO study protocol paper.^25^ The severity of M-D was assessed using the clinical global impression severity (CGI-S) scale by R.M. and E.T., where the mean of both scores was used as the final score for the analysis. The CGI-scores ranged from 1 (normal, no movement disorder) to 7 (among the most extremely affected patients). Additionally, patients self-rated the severity of their movement disorder on a Visual Analogue Scale. With regard to non-motor symptoms, the severity of anxiety and depression was assessed using the Hospital Anxiety and Depression Scale (HADS), and the Montreal Cognitive Assessment (MoCA) was used to screen for cognitive impairment. While non-motor symptoms are clinically relevant and recognized as important features of the disease, they were not the primary focus of this study. Instead, HADS and MoCA scores were included as covariates in the connectivity analyses to control for individual differences in psychiatric and cognitive symptomatology. This approach was chosen to minimize the potential confounding effects of non-motor symptoms on brain connectivity patterns implicated in the various levels of motor control.

### Clinical and demographic data analysis

Analyses were performed using the Statistical Product and Service Solutions (SPSS, version 28, IBM Corp., Armonk, NY). Normality of continuous variables was assessed by using the Shapiro-Wilk test and through visual inspection of the histograms of the respective distributions. In the case of non-normality, non-parametric tests were used to compare group estimates.

### MRI acquisition

MRI data were collected on a 3T Siemens Prisma scanner at the UMCG using a Siemens 64-channel head coil. High-resolution (MPRAGE) T1-weighted images were acquired using the following parameters: TR: 2300 ms; TE: 2.98 ms; FOV: 256; FOV Phase: 93.8%; FA: 9°; 176 sagittal slices with 1 mm thickness. Full brain, multi-band rsfMRI scans were acquired using a T2*-weighted echo-planar sequence with the following scanning parameters: TR = 1600 ms; TE = 34 ms; FOV = 224 mm; FA = 70°; voxel size = 2 mm isotropic; 72 slices; partial Fourier = 6/8; MB = 4; bandwidth = 1828 Hz/px; pulse duration = 5120 µs; AP phase encoding direction; MB LeakBlock kernel enabled; EPI factor = 114, for a total duration of 9 min and 52 s. In addition, 10 volumes with inverted RO/PE polarity were acquired for AP-PA distortion correction purposes. The sequence was generously provided by the Center for Magnetic Resonance Research (CMRR) at the University of Minnesota. For the rsfMRI scan, participants were instructed to lie motionless in the scanner, fixate on a white fixation cross displayed on a black screen and let their minds wander freely.

### Preprocessing of MRI data

MRI data was preprocessed using *fMRIPrep* version v22.0.2,^26^ which is based on *Nipype 1.7.0*,^27^ a flexible neuroimaging data processing framework in *Python*. Automatic removal of motion artefacts using independent component analysis (ICA-AROMA) was performed on the preprocessed BOLD in MNI space time-series after removal of non-steady state volumes and spatial smoothing with a Gaussian kernel of 6 mm full-width half-maximum.^28^

### MRI data quality and motion assessment

To address potential motion-induced bias, especially relevant in studies involving hyperkinetic movement disorders, we conducted a multi-step quality control procedure. First, all raw fMRI datasets underwent a visual assessment of images on an individual basis, supplemented by reports generated by MRIQC and fMRIPrep (automatically generated when running the fMRIprep pipeline). This included checking for artefacts, signal dropout and excessive motion. Additionally, we reviewed the fMRIPrep preprocessing outputs in detail, including the HTML quality and ICA-AROMA reports. This review was conducted independently by two authors (R.M. and J.D.). All 19 patient scans were deemed usable based on predefined quality criteria, which included the absence of major artefacts, successful co-registration and adequate preprocessing. No participants were excluded from analysis based on motion-related artefacts.

To quantify head motion, we used the framewise displacement (FD) values provided in the MRIQC reports for each subject. These FD values were compared between patients and healthy volunteers to assess potential group differences in motion, which yielded no significant differences between groups.

### Independent component analysis

This analysis aims to investigate the BGTC and CTC brain networks in patients with M-D compared to healthy volunteers during rest. To investigate brain networks from the rsfMRI data, we first computed brain network components with group-ICA using a spatially constrained independent component analysis (scICA) in the Group ICA of fMRI toolbox (GIFT; version 4.0c).^29-31^ This algorithm utilizes template components as reference signals to extract only desired sources (brain networks), in this case.

Fifty-three networks (i.e. independent components) from the NeuroMark framework were used as templates for scICA, resulting in 53 subject-specific networks (each consisting of a spatial map and a time course) per person.^32^ Values of each subject’s component image and time course were converted to *Z*-scores. Subject-specific time courses were detrended and despiked using AFNI’s 3dDespike,^33^ then filtered using a fifth-order Butterworth low-pass filter with a high frequency cutoff of 0.15 Hz.

### Network (component) selection

**Figure 1:**
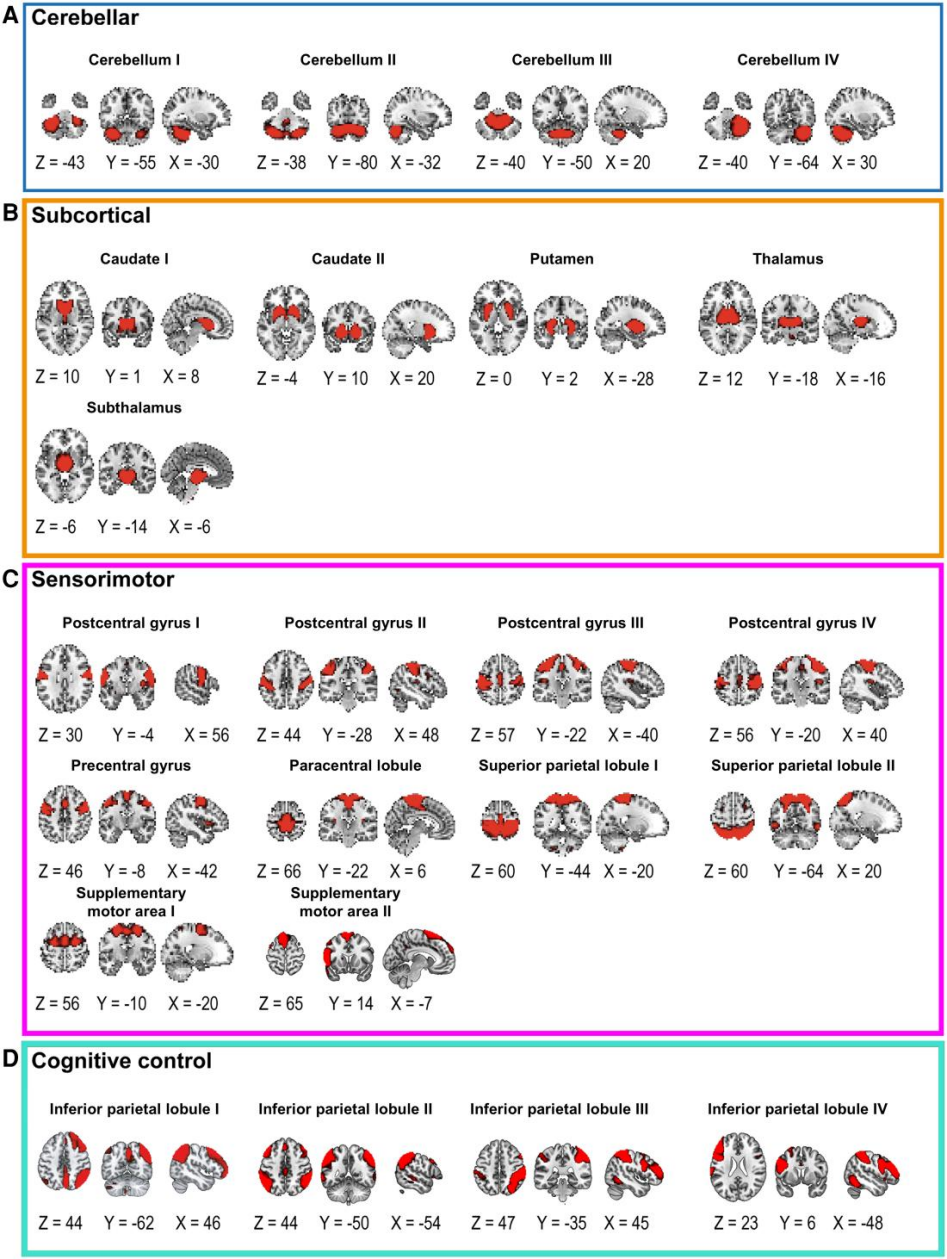
**Intrinsic connectivity networks considered for the static and dynamic FC analysis.** Spatial maps of the 23 networks (i.e. independent components) computed for the entire sample (19 M-D patients and 19 healthy volunteers) are shown, thresholded at *Z*-score > 1. These networks are classified into four higher-order domains: cerebellar (**A**, four components), subcortical (**B**, five components), sensorimotor (**C**, 10 components) and cognitive control (**D**, 4 components).

To investigate how the BGTC and CTC networks are functionally connected, we selected 23 network components encompassing these networks. These networks can be grouped into 4 functional domains: 10 sensorimotor, 4 cognitive control, 5 subcortical (including basal ganglia and thalamus) and 4 cerebellar (Fig. 1). While the NeuroMark atlas officially defines nine sensorimotor networks, we included a 10^th^ network—the supplementary motor area—in our sensorimotor domain. Although the SMA is categorized under the cognitive-control domain in the NeuroMark framework, given its well-established role in motor planning and execution, we included it in the sensorimotor domain for this study. Furthermore, of the 17 components in the cognitive control domain in the NeuroMark atlas, we selected four components that include parietal regions to capture potential functional alterations relevant for higher-order motor control. This targeted selection was informed by prior literature indicating the involvement of the parietal cortex, particularly the inferior parietal lobule, in motor dysfunction observed in conditions such as M-D. For a complete overview of these brain networks and peak MNI coordinates, see Supplementary Table 1.

### Static functional connectivity analysis

We aimed to examine static within-network and between-network FC in patients with M-D, which is related to the connectivity within a network and connectivity between networks, respectively.^34^ We chose to analyse both these connectivity measures as they are distinct but complementary facets of FC, coupled with the fact that M-D pathology might manifest within networks rather than between networks, and vice versa. For this analysis, we used the MANCOVAN toolbox in the GIFT software (version 1.0).

‘Within-network’ FC was evaluated using the subject-specific spatial maps of the 23 included network components. Analyses were performed on the signal intensities of the respective spatial maps. These intensities indicate the spatial contribution of voxels to the average network signal time course map.^34^ Spatial maps were thresholded to focus the analysis on the subgroup of voxels most representative of each network. Thresholding was based on the distribution of voxelwise *t*-statistics to identify voxels with consistent and high activation (t > mean + 4SD) across all subjects.^34^ ‘Between-network’ FC was assessed by computing Pearson’s correlation coefficient on *Z*-transformed time courses for each participant.^35^ We applied false discovery rate (FDR) correction for multiple comparisons with the statistical threshold for significance set at *P*_fdr_ < 0.05. For within-network FC, this correction was applied to each spatial map separately; for between-network FC, it was applied to the entire set of component pairs. To additionally address multiple testing for the included spatial maps, i.e. across 23 components, we concatenated all *P*-values from the univariate analyses across components per domain and performed FDR correction in toto per domain.

In addition to investigating differences between M-D patients and healthy volunteers, a within-group MANCOVA model was used to test whether static connectivity is related to the motor severity of M-D patients, as measured using the CGI scale.

### Dynamic functional connectivity analysis

#### Average sliding window approach

In addition to static intra-network and inter-network FC, we also aimed to investigate dynamic FC in patients with M-D. A commonly adopted strategy to examine dynamic FC is a sliding window correlation approach.^21^ In this study, we computed dynamic FC using the average sliding window correlation (ASWC) approach as implemented in GIFT (version 3.0b).^36^ We opted for this approach, given its advantages in estimating dynamic FC compared to the standard sliding window approach. The ASWC approach involves averaging a predetermined number of consecutive sliding window correlations, progressing one step at a time and repeating the process. In contrast to the standard sliding window approach, where only the window length needs to be specified, the ASWC approach requires setting both the window length and the averaging length. The advantages of this approach include the use of a smaller window length, which allows for better tracking of temporal dFC fluctuations, fewer artefact fluctuations and its function as a high-pass filter, aiding in the removal of nuisance frequencies (Vergara *et al*., 2019).

In line with previous studies, resting-state data were analysed using sliding windows of 28 repetition times (44 s), yielding 337 windows per subject.^36-38^ Successive windows were shifted in steps of one repetition time each, which means that windows are overlapping. Fisher’s z-transformation was then applied to these correlation matrices. Given that we selected 23 network components for further analysis, each window consisted of 23 × 22/2 = 253 Pearson correlations. Subsequently, ASWC [window size = 31 repetition times (50 s)] were calculated for every subject, resulting in 306 windows per subject.^36^

#### Clustering analysis

Aimed at identifying recurring patterns of network FC, a *k*-means clustering procedure (maximum iterations = 5000; number of replicates = 42) was applied to ASWC to detect different connectivity states.^38^ We used the correlation distance metric to estimate the similarity between ASWC matrices.^39^ The clustering approach was applied twice: first, to determine the optimal number of clusters *k* (connectivity states) and then to construct the actual *k* connectivity states. To estimate the optimal number of clusters, we used the elbow criterion, computed as the ratio between within-cluster distances to between-cluster distances,^38^ and the silhouette measure,^40^ following previous studies.^38,39,41,42^ This was done for a range of *k* (2–10) using subject-specific local maxima in variance of the ASWC (subject exemplars), i.e. windows showing the highest variance. Furthermore, the minimum frequency of states was set at 10% (i.e. across all participants, at least 10% of windows needed to be assigned to a specific state).

The elbow criterion indicated a cluster size of four, while the silhouette measure indicated two clusters (Supplementary Fig. 1). Based on these results, we opted for a cluster size in between these estimates, namely a three-cluster model. Each window of each participant was assigned to one of the connectivity states. Notably, this analysis does not guarantee that all participants visit all of these connectivity states.^41^ That is, some might enter only one or two states, despite there being three connectivity states. Given the difference in cluster estimations between the elbow and silhouette measures, we additionally performed sensitivity analyses using a two- and four-cluster model to determine whether results remained similar. We also performed a sensitivity analysis whereby we considered all brain network components instead of the 23 networks of interest to examine our results when using a broader scope of brain regions. These sensitivity analyses were conducted to ensure the robustness and generalizability of our findings.

#### Analysis of state summary measures

We investigated the following state summary measures as indicators of dynamic connectivity: (i) mean dwell time (the time a participant spent on average in a given state without switching to another one; (ii) fraction of time spent in every state (percentage of total time a participant spent in a certain state); (iii) number of visits per state (amount of times a participant visited a specific state); and (iv) number of transitions between states. Similar to the static connectivity analysis, we also examined whether state summary measures are related to the severity of motor symptoms in M-D patients. We applied FDR correction for multiple comparisons (three states) with the statistical threshold set at *P*_fdr_ < 0.05.

## Results

### Demographics and clinical characteristics

A Shapiro-Wilk test showed a significant departure from normality for all continuous variables (*P* < 0.05). Accordingly, these variables are reported as median [interquartile range (IQR)]. In agreement with the selection of healthy volunteers, there were no significant differences in age, sex and handedness between M-D patients and healthy volunteers (Table 1).

**Table 1:** Patient characteristics

	M-D (*n* = 19)	Healthy (*n* = 19)	*P*-value
**Age, median (IQR), years**	27 (39)	28 (32)	0.58
**Sex, males/females**	16/3	15/6	0.45
**Age of onset (IQR), years**	9 (9)	NA	NA
**Disease duration, median (IQR) years**	21 (40)	NA	NA
**CGI-S Score (0–7), median (IQR)**	3 (2)	NA	NA
**SGCE-positive**	10	NA	NA
SGCE mutation inheritance, father/mother*	7/1	NA	NA
**Handedness, right/left**	17/2	14/5	0.40
**MOCA (IQR)**	26.00 (4.00)	28.69 (2.50)	<0.001
**HADS anxiety (IQR)**	6.78 (6.00)	2.81 (5.00)	<0.05
**HADS depression (IQR)**	3.42 (5.00)	1.19 (2.00)	<0.05

Abbreviations: CGI-S, Clinical Global Impression-Severity Scale; NA, not applicable; *SGCE*, epsilon-sarcoglycan; data are presented as the mean ± SD unless specified otherwise.

^*^Two patients had *de novo SGCE* mutations. Most frequently affected body parts in the M-D group included the arms (58%), neck (37%) and trunk (26%). 26% of M-D patients had a generalized phenotype.

### Static functional connectivity

**Figure 2:**
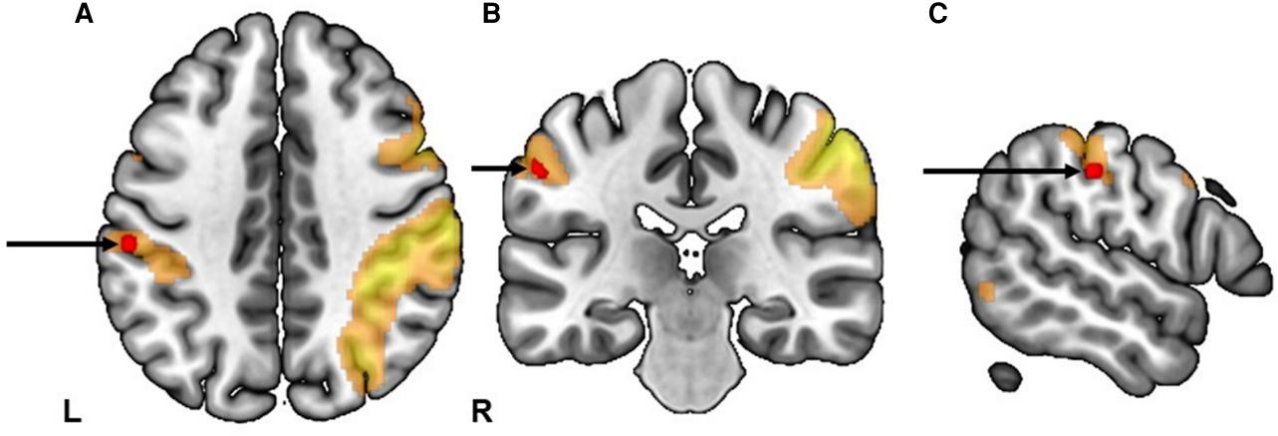
**Static FC analysis.** Within-network FC was evaluated using the signal intensities of subject-specific spatial maps, which were first thresholded [t > mean + 4SD (standard deviation)] to focus on voxels most representative of each network. Multivariate analysis of covariance (MANCOVAN) was used to compare 19 M-D patients and 19 healthy volunteers. Results are displayed in (**A**) axial, (**B**) coronal and (**C**) sagittal views. M-D patients exhibited increased within-network FC (arrow) in an extended cognitive control network (highlighted region) compared to healthy volunteers (*P*_fdr_ < 0.05). This difference was observed in the left inferior parietal lobule. For visualization purposes, results are shown thresholded at *P* = 0.001 (uncorrected).

‘Within-network’ MANCOVAN analyses showed a statistically significant difference in a cognitive control network (inferior parietal lobule III) spatial map (Fig. 2). Specifically, within-network FC was increased in the anterior segment of the left supramarginal gyrus (*P*_fdr_ < 0.05) for M-D patients. However, when applying FDR correction to all voxel-wise *P*-values of the univariate analyses across components within the cognitive control domain, no significant clusters were detected. There were no significant differences between groups ‘in between-network’ FC. Furthermore, we found no relation between static connectivity and motor symptom severity in M-D patients. These results remained similar after adjusting for HADS and MOCA scores in the analysis.

### Dynamic connectivity states

**Figure 3:**
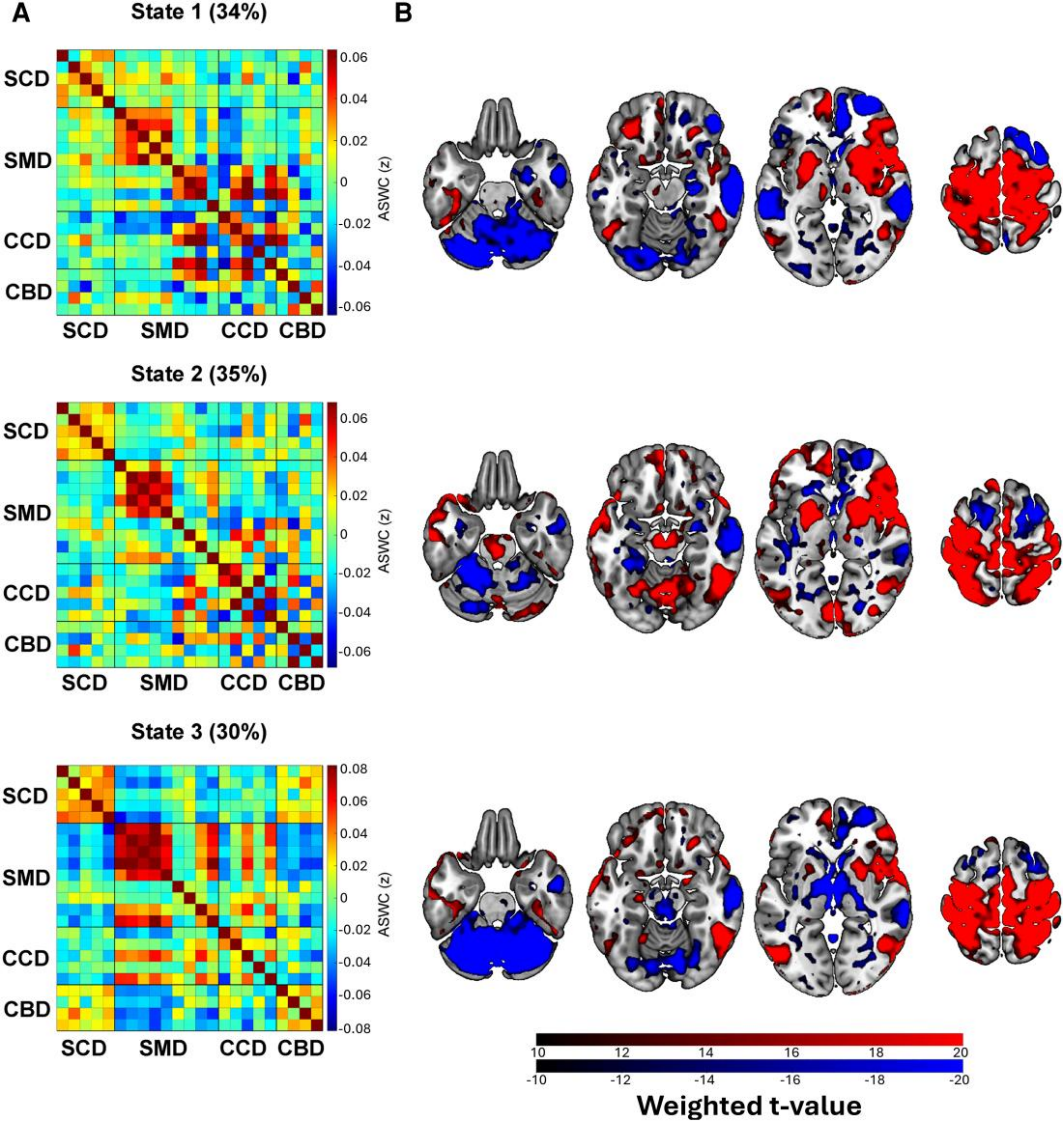
**State centroids (i.e. mean dFC that form the center point of a cluster) for every state calculated for the entire study sample.** (**A**) Connectivity matrices of three states are shown, using dFC data from 19 M-D patients and 19 healthy volunteers. The majority of all windows was assigned to State 1 (34%), followed by State 2 (35%) and State 3 (30%). (**B**) Rendered brain images depict the independent components’ *t*-maps weighted by the sum of ASWC for every component in the centroid (i.e. sum of a column or row). Red colours represent positive voxels, blue negative. ASWC, average sliding window correlation; CBD, cerebellar domain; CCD, cognitive control domain; dFC, dynamic functional connectivity; SCD, subcortical domain; SMD, sensorimotor domain.

By applying *k*-means clustering, we identified three prototypical dynamic connectivity states in the BGTC and CTC circuits. Figure 3 shows the three state centroids (i.e. mean pattern of dynamic connectivity for states). State 1 is characterized by a mixture of strong positive and negative connectivity between sensorimotor and cognitive control domains. Within the subcortical domain, there is mostly positive connectivity, while in the sensorimotor and cerebellar domains, there is a mixture of positive and negative connectivity between networks. In the sensorimotor domain, there are two segregated subdomains visible: (i) connectivity between the postcentral gyrus, paracentral lobule and superior parietal lobule networks and (ii) between the superior parietal lobule, paracentral lobule, postcentral and precentral gyrus networks. State 2, characterized by relatively less pronounced anticorrelations between the sensorimotor and cognitive control domains, did not feature two clear segregated subdomains in the sensorimotor domain, as observed in State 1. State 3 is characterized by strong segregation of the sensorimotor domain from the subcortical and cerebellar domains, while there is strong connectivity between networks within these domains.

State 2 was the most frequently occurring state across all subjects (35%), followed by State 1 (31%) and State 3 (30%). Notably, not all participants visited all three connectivity states in their BGTC and CTC circuits. Furthermore, some participants remained in one connectivity state during the entire resting-state scan.

### State summary measures

**Figure 4:**
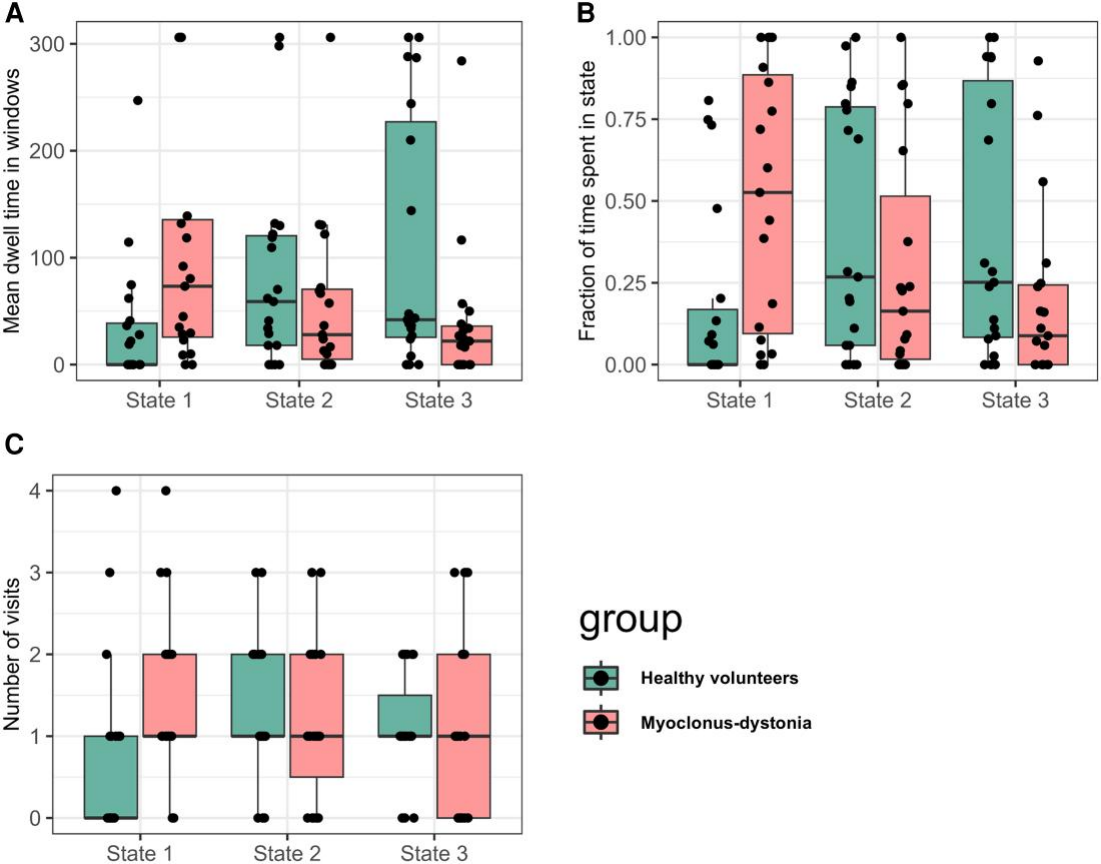
**State summary measures.** Four state summary measures were investigated for the three identified connectivity matrices (using k-means clustering): mean dwell time, fraction of time spent, number of visits and number of transitions. Group differences were evaluated using Mann-Whitney U-tests and FDR-correction for multiple comparisons (three states) was applied with the statistical threshold set at *P*_fdr_ < 0.05. (**A**) Mean dwell times. M-D patients spent significantly more time in State 1 and less time in State 3 at any one time (*P*_fdr_ < 0.05). (**B**) Fraction of times spent in brain states for M-D and HC groups. Over the entire scan, M-D spent significantly more time in State 1 than healthy volunteers (*P*_fdr_ < 0.05). In addition, M-D spent significantly less time in State 3 compared to healthy volunteers (*P*_fdr_ < 0.05). (**C**) Number of visits. Patients with M-D visited State 1 significantly more often compared to healthy volunteers (*P*_fdr_ < 0.05).

We subsequently tested for between-group differences (patients against healthy volunteers) in state summary measures. As reported in Fig. 4, we found significant group differences in the mean dwell time and fraction of time spent in States 1 and 3. Specifically, the M-D group spent more time in State 1 than healthy volunteers and had a longer dwell time in this state once they entered it (*P*_fdr_ < 0.05). In contrast, M-D patients spent less time in State 3 and had a shorter dwell time in this state (*P*_fdr_ < 0.05). In addition, patients with M-D visited State 1 significantly more often compared to healthy volunteers. Moreover, we found no significant differences between groups in the number of transitions between states. Finally, we found no relationship between state summary measures and motor symptom severity in M-D patients. These results remained similar after adjusting for the HADS and MOCA scores in the analysis.

We repeated the main dynamic connectivity analysis steps using a two-cluster and four-cluster model as well as a whole-brain model. Supplementary Figs 2–4 show the results of these sensitivity analyses. The results of these sensitivity analyses largely align with the results reported in the main analysis.

## Discussion

We employed independent component analysis (ICA), followed by a within- and between-network static FC analysis. In addition, we also applied a clustering procedure to detect different dynamic connectivity states. Static connectivity analyses revealed that M-D patients had increased connectivity of the left supramarginal gyrus within an (antero-)inferior parietal lobule network of the cognitive control domain, when correcting for voxel-wise FDR at the level of this network only. With this analysis, no changes were seen in the connectivity profiles of the cerebellum and basal ganglia, respectively. Dynamic FC analysis showed that patients with M-D moved away from a configuration characterized by high segregation of basal ganglia and cerebellum from sensorimotor networks, along with high connectivity between networks (independent components) within these domains. Reduction of time in such a temporal state dominated by high intrinsic basal ganglia and cerebellar connectivity may fit with impaired motor preparation, leading to more disorganized movements, i.e. myoclonus and dystonia. Our study thus points to large-scale functional reorganization of both the BGTC and CTC networks in M-D.

Dynamic analyses revealed that M-D patients spent less time in State 3, a brain state characterized by relatively high intrinsic connectivity within the basal ganglia and cerebellum and functional segregation from more distant network regions. This finding could suggest diminished local neuronal processing within subcortical structures in M-D patients. Less segregation of basal ganglia and cerebellar processing preceding cortical motor output may indicate a decreased preparatory contribution to the sensorimotor cortical network.^43,44^ Although we cannot demonstrate a temporal order between brain states, the concept of distinguishing between preparation and execution allows for the assumption of a sequence in time. In M-D, this appears to result in disorganized movement, manifesting as myoclonus and dystonia. There was a reduced presence in State 3, but this was contrasted by a higher occurrence of State 1. This state is characterized by high intrinsic connectivity within the sensorimotor domain, though this is relatively restricted to the precentral gyrus, paracentral and superior parietal lobule networks. Moreover, connectivity of the sensorimotor domain with other domains showed a mixed pattern, including less uniformly organized connections with the cognitive domain. This may increase the risk of aberrant motor output without context or purpose, leading to unwanted movements. An alternative interpretation to consider is that the increased time spent in State 1 reflects a compensatory mechanism, whereby patients attempt to exert greater control over their movements during scanning. While this is a plausible explanation, the reduced presence in State 3—alongside its impaired cerebellar (extrinsic) network contribution—remains central to our interpretation of a network imbalance that increases susceptibility to cortical ‘hyperactivity’. This view is further supported by the anterior parietal effects seen in the static functional connectivity analysis and is consistent with cerebellar involvement reported in previous dystonia literature.

The intrinsic connectivity effects within the sensorimotor domain identified by the dynamic analysis align with the static analysis, which revealed increased connectivity within the anterior segment of the left supramarginal gyrus in M-D patients. However, as this effect did not remain significant after correction across all components within the cognitive control domain, it should be interpreted with caution. The underlying heightened activity, particularly in this part of the cognitive control domain, may be due to inefficient interaction with the cerebellum regarding its feed-forward function in motor planning. While the dorsal surface of the supramarginal gyrus along the intraparietal sulcus is associated with motor intention,^45-47^ the anterior wall along the postcentral sulcus, transitioning to the postcentral gyrus, is involved in processing proprioceptive information to guide effective movement.^48-50^ This cortical region integrates somatosensory and intentional aspects of motor control, with a crucial role of the cerebellum in processing the predicted sensory consequences of intended movement.^51-53^ Since this ‘sensory’ component in the temporal planning of movements is a basic constituent of motor control, dysfunction due to an imbalance in cerebellar-parietal interaction might contribute to the irregular release of unwanted movements seen in M-D patients, indeed suggesting the involvement of a common neural mechanism involved in myoclonus and dystonia.

This explanation of inefficient cortico-cerebellar interaction aligns with a recent study by Latorre and colleagues (2024).^54^ They described that transcranial cerebellar stimulation enhanced sensorimotor cortex excitability in patients with cortical myoclonus, while no effect was seen in healthy subjects. Contrary to expectations, an inhibitory effect was not observed in that study. Additionally, such stimulation resulted in increased somatosensory evoked potentials (SEP) in patients with giant SEPs, explained as an indicator of cortical reorganization due to altered cerebellar interconnections.^54^ The effect of cerebellar stimulation on the SEP in cortical myoclonus indicates that altered sensory processing appears to play a role in the underlying pathophysiology. Despite the subcortical origin of myoclonus in M-D, this sensory aspect may highlight an interesting equivalence between myoclonus and dystonia. As explained above, we propose that altered sensory processing may particularly concern the impaired prediction of sensory consequences of movement. In dystonia, the impact of sensory processing on motor planning is shown by a sensory trick, which can alleviate the dystonic posture, adding support to the hypothesis of cerebellar involvement in dystonia pathophysiology.^55^ In focal dystonia, cortical changes have also been described in particular parietal and sensorimotor regions, reflecting more widely distributed functional network changes (Battistella *et al*., 2017). In this respect, it’s worth noting that the main type of dystonia in the M-D study cohort was also focal dystonia, together with myoclonus.

Importantly, we found no relationship between static and dynamic connectivity measures and symptom severity. This may be because M-D tends to manifest more prominently during tasks. Therefore, resting-state fMRI may not effectively capture the neural activity associated with task-related symptom severity. Additionally, the median symptom severity score of M-D patients was relatively low, which might not be associated with disrupted connectivity patterns, coupled with the fact that this study might be underpowered to find such effects. On the other hand, our results pertain to subtle network changes that likely include compensatory adjustments. This introduces an effect that may not be adequately captured using a linear severity scale.

### Further considerations

It should be noted that the patients in our cohort, regardless of mutation status, shared a largely similar clinical phenotype, characterized by prominent myoclonus and dystonia, primarily affecting the upper body. This phenotypic overlap formed the basis for analysing them as a single group, in line with previous literature. For example, Roze *et al*. (2018) proposed that mutation-positive and mutation-negative cases can represent a broader, shared MD phenotype, which supports combining them in studies focusing on motor network dysfunction. Nonetheless, clinical and pathophysiological differences may exist between SGCE-positive and SGCE-negative patients, which is an important consideration for future studies.

A further important consideration is the clinical assessment of symptom severity. In this study, we used the Clinical Global Impression–Severity (CGI-S) scale to rate overall motor symptom severity. While this scale provides a general indication of clinical burden, it is a relatively coarse measure with only seven scoring points and does not differentiate between rest and action symptoms. Given that symptoms in MD are typically more pronounced during action, the CGI-S may be disproportionately influenced by these features. Moreover, the majority of patients in our cohort scored relatively low on this scale, reflecting mild to moderate symptom severity overall. Ideally, more specialized rating scales—such as the Burke-Fahn-Marsden Dystonia Rating Scale (BFMDRS) for dystonia and the Unified Myoclonus Rating Scale (UMRS) for myoclonus—would allow for a more granular and symptom-specific evaluation. However, even these tools have limitations when applied to MD, especially given the often mixed and mild symptom presentation. As discussed by Zutt *et al*. (2016), existing scales may not fully capture the clinical heterogeneity seen in genetically confirmed MD populations.^56^ These limitations should be taken into account when interpreting the clinical correlations with imaging findings. Future studies may benefit from the development or adoption of MD-specific rating instruments and from examining the relationship between symptoms during rest and resting-state neural activity more directly.

## Conclusion

Taken together, this combined assessment of static and dynamic connectivity traits in patients with M-D demonstrated disrupted brain network dynamics in the BGTC and CTC circuits associated with motor control, distinct from those observed in healthy volunteers. This aligns with the hypothesis of compromised basal ganglia and cerebellar network functioning in this disorder. Specifically regarding the involvement of the cerebellum, our findings support the model that pathology-induced intra-cerebellar network changes lead to functional alterations in cortical regions critical for sensory-motor integration. Impairment in these regions may consequently result in the release of uncontrolled myoclonic and dystonic movements. Future neuroimaging and electrophysiological studies that (i) involve tasks reliant on both basal ganglia and cerebellum function and (ii) assess the temporal order in functional network dynamics may provide further insight into the causal link between dysfunction in these regions and M-D.

## Supplementary Material

fcag205_Supplementary_Data

## Data Availability

Anonymized processed datasets generated and/or analysed in the current study are available from the corresponding author upon reasonable request from qualified investigators after permission of the appropriate regulatory bodies.
