# Supplementary material for: Altered motor network dynamics in myoclonus-dystonia
Source: Brain Commun. 2026 Jul 21;8(4):fcag205. doi: 10.1093/braincomms/fcag205 (PMC13386016; doi:10.1093/braincomms/fcag205)
Supplement: fcag205_Supplementary_Data [file fcag205_supplementary_data.docx]

# **Supplementary material**

**B**

**A**


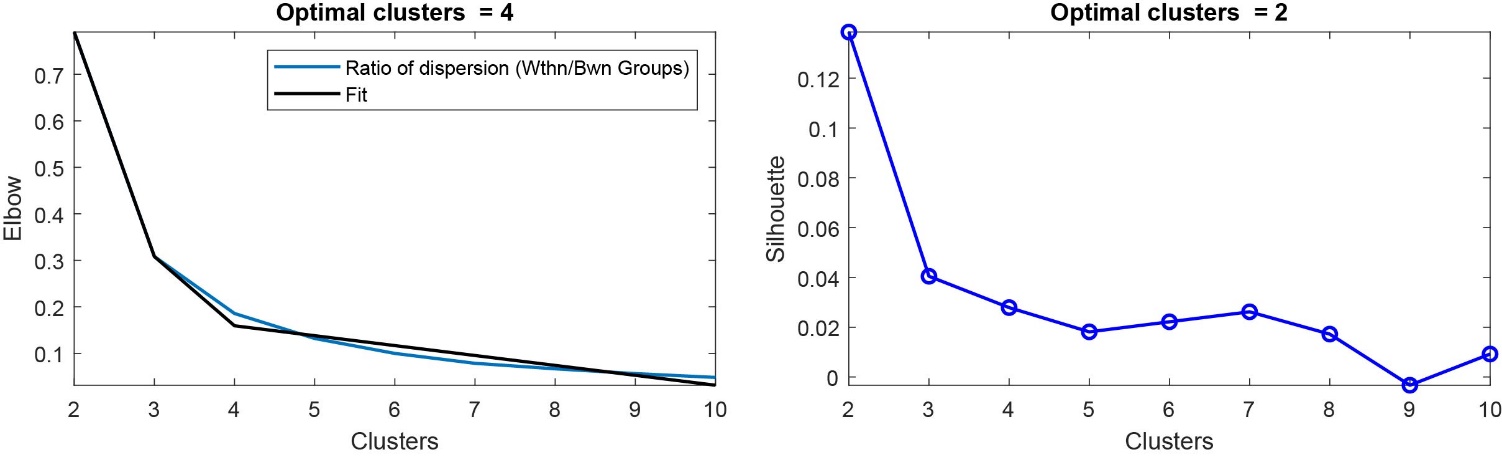


**Supplementary Figure 1. Results of the cluster estimation using the elbow criteria and silhouette measure.** Bwn = between, Wthn = within.


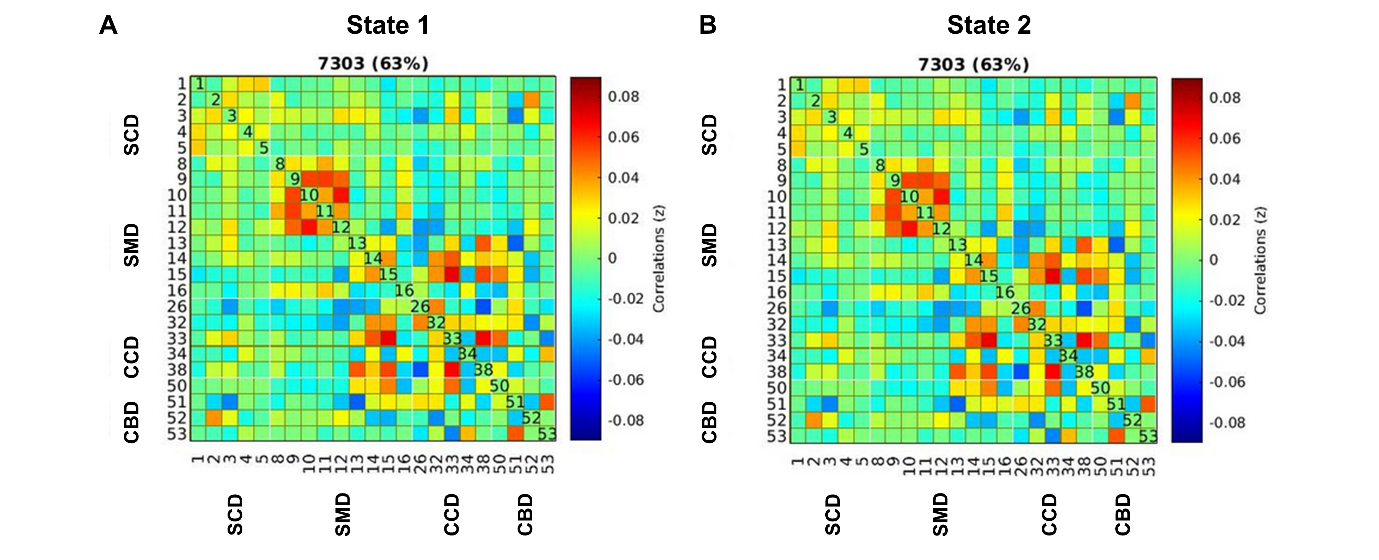


**Supplementary Figure 2. Sensitivity analysis sensitivity analyses using a 2-cluster model to compare these results with the main analysis (Fig. 3).** Centroids (i.e., mean dFC which form the center point of a cluster) for every state calculated for the entire study sample. Two connectivity states are shown. The majority of all windows was assigned to State 1 (63%), followed by State 2 (37%). State 1 resembled a combination of State 1 and State 2 observed in the main analysis, whereas State 2 showed a high resemblance to State 3. In this 2-cluster model, differences were identified in the mean dwell time and fraction of time spent only in one state as opposed to two states observed in the main analysis. Specifically, M-D patients spent less time in State 2 and dwelled shorter in this state, similar to the finding for State 3 in the main analysis (P*_fdr_*<0.05). CBD = cerebellar domain; CCD = cognitive control domain; SCD = subcortical domain; SMD = sensorimotor domain.


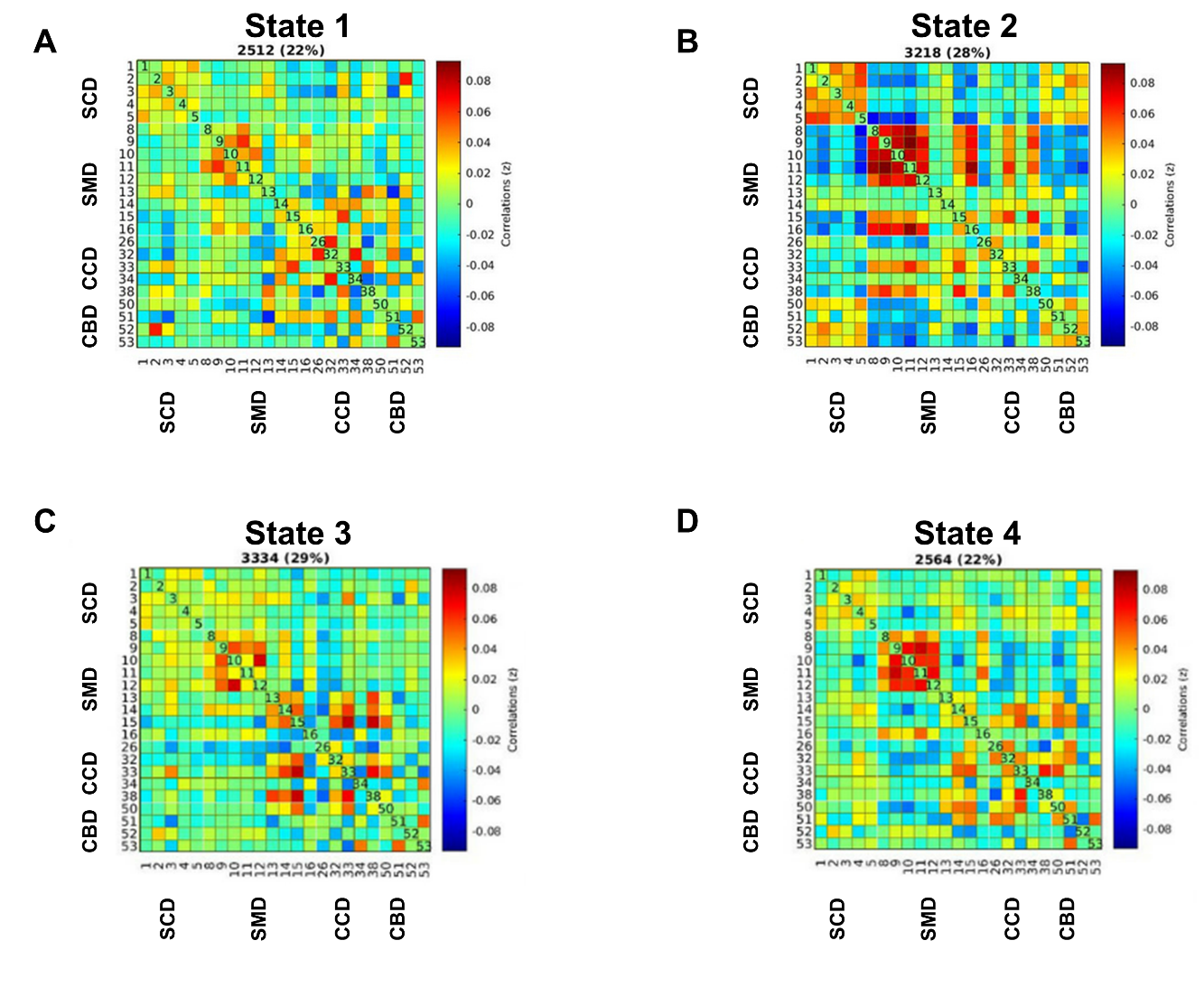
**Supplementary Figure 3. Sensitivity analysis sensitivity analyses using a 4-cluster model to compare these results with the main analysis (Fig. 3).** Centroids (i.e., mean dFC which form the center point of a cluster) for every state calculated for the entire study sample. Four connectivity states are shown. The majority of all windows was assigned to State 3 (29%), followed by State 2 (28%), State 4 (22%), and State 1 (22%). State 3 most resembled State 1 observed in the main analysis, State 2 showed a high resemblance to State 3, State 4 resembled State 2, whereas State 1 likely represents a combination of State 1 and 2 from the main analyis. In this 4-cluster model, differences were identified in the mean dwell time and fraction of time of two states, similar to the main analysis. Specifically, the M-D group spent more time in State 3 in total as well as dwelled longer in this state once having entered it, and spent less time in State 2 and dwelled shorter in this state, similar to the findings described in the main analysis (P*_fdr_*<0.05). CBD = cerebellar domain; CCD = cognitive control domain; SCD = subcortical domain; SMD = sensorimotor domain.


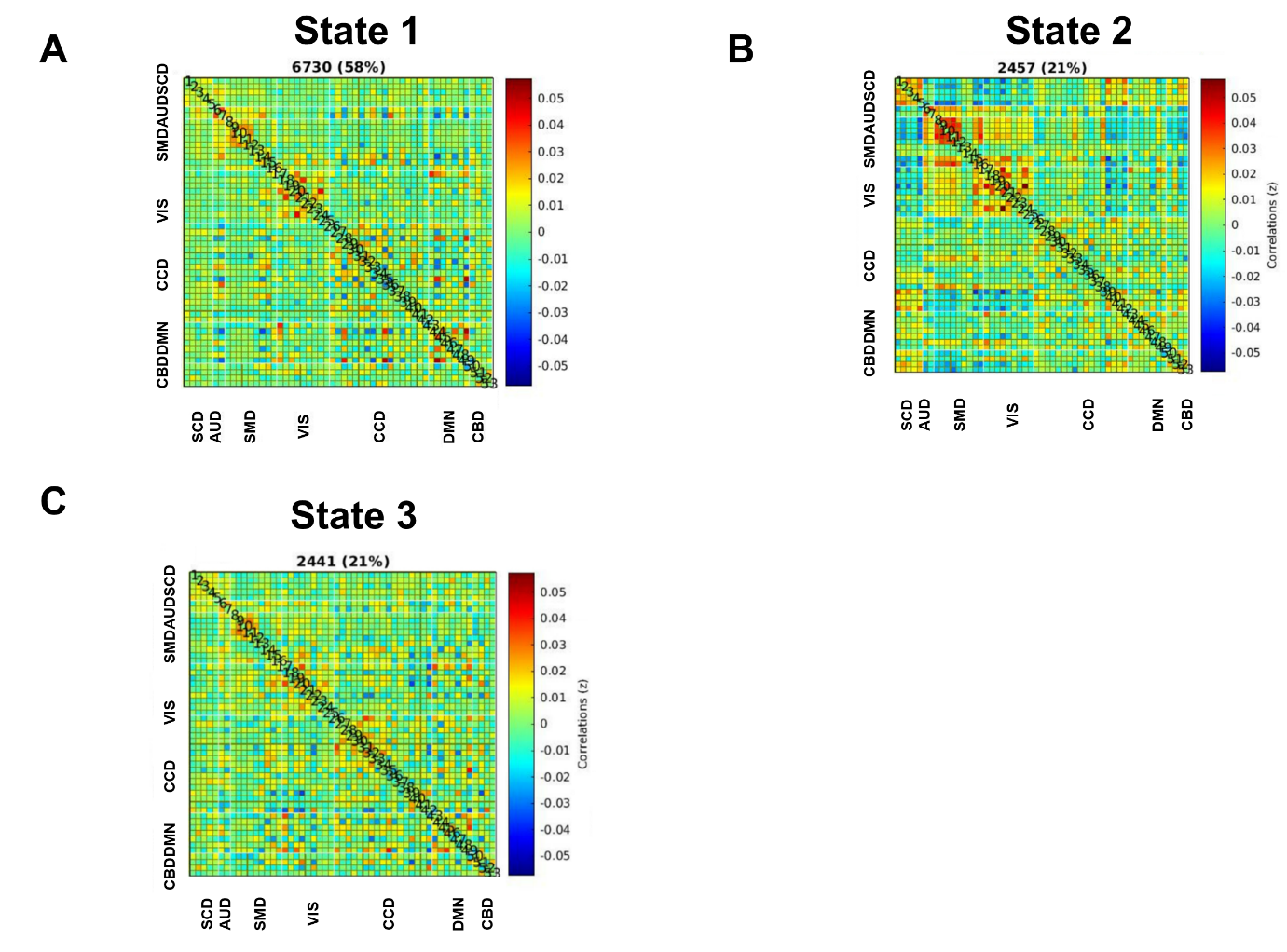


**Supplementary Figure 4. Sensitivity analysis sensitivity analyses using a 3-cluster whole-brain model to compare these results with the main analysis (Fig. 3).** Centroids (i.e., mean dFC which form the center point of a cluster) for every state calculated for the entire study sample. Three connectivity states are shown. The majority of all windows was assigned to State 1 (58%), followed by State 2 (21%), and State 3 (21%). States 1 and 3 are similar to States 1 and 2 from the main analysis, whereas State 2 shows a high resemblance to State 3 from the main analyis. In this 3-cluster whole-brain model, differences were identified in the mean dwell time and fraction of time of two states, similar to the main analysis. Specifically, the M-D group spent more time in State 1 in total as well as dwelled longer in this state once having entered it (P*_fdr_*<0.05), and spent less time in State 2 and dwelled shorter in this state, similar to the findings described in the main analysis (trend: P*_fdr_*=0.09). AUD = auditory domain; CBD = cerebellar domain; CCD = cognitive control domain; DMN = default mode network domain; SCD = subcortical domain; SMD = sensorimotor domain; VIS = visual domain.

Supplementary Table 1. NeuroMark networks.

| **Selected Components as Regions of Interest** | **X** | **Y** | **Z** |
| --- | --- | --- | --- |
| **Subcortical network (SC)** | | | |
| Caudate (1) | 6.5 | 10.5 | 5.5 |
| Subthalamus/hypothalamus (2) | -2.5 | -13.5 | -1.5 |
| Putamen (3) | -26.5 | 1.5 | -0.5 |
| Caudate (4) | 21.5 | 10.5 | -3.5 |
| Thalamus (5) | -12.5 | -18.5 | 11.5 |
| **Auditory network (AUD)** | | | |
| Superior temporal gyrus ([STG], 6) | 62.5 | -22.5 | 7.5 |
| Middle temporal gyrus ([MTG], 7) | -42.5 | -6.5 | 10.5 |
| **Sensorimotor network (SM)** | | | |
| Postcentral gyrus ([PoCG], 8) | 56.5 | -4.5 | 28.5 |
| Left postcentral gyrus ([L PoCG], 9) | -38.5 | -22.5 | 56.5 |
| Paracentral lobule ([ParaCL], 10) | 0.5 | -22.5 | 65.5 |
| Right postcentral gyrus ([R PoCG], 11) | 38.5 | -19.5 | 55.5 |
| Superior parietal lobule ([SPL], 12) | -18.5 | -43.5 | 65.5 |
| Paracentral lobule ([ParaCL], 13) | -18.5 | -9.5 | 56.5 |
| Precentral gyrus ([PreCG], 14) | -42.5 | -7.5 | 46.5 |
| Superior parietal lobule ([SPL], 15) | 20.5 | -63.5 | 58.5 |
| Postcentral gyrus ([PoCG], 16) | -47.5 | -27.5 | 43.5 |
| **Visual network (VIS)** | | | |
| Calcarine gyrus ([CalcarineG], 17) | -12.5 | -66.5 | 8.5 |
| Middle occipital gyrus ([MOG], 18) | -23.5 | -93.5 | -0.5 |
| Middle temporal gyrus ([MTG], 19) | 48.5 | -60.5 | 10.5 |
| Cuneus (20) | 15.5 | -91.5 | 22.5 |
| Right middle occipital gyrus ([R MOG], 21) | 38.5 | -73.5 | 6.5 |
| Fusiform gyrus (22) | 29.5 | -42.5 | -12.5 |
| Inferior occipital gyrus ([IOG], 23) | -36.5 | -76.5 | -4.5 |
| Lingual gyrus ([LingualG], 24) | -8.5 | -81.5 | -4.5 |
| Middle temporal gyrus ([MTG], 25) | -44.5 | -57.5 | -7.5 |
| **Cognitive-control network (CC)** | | | |
| Inferior parietal lobule ([IPL], 26) | 45.5 | -61.5 | 43.5 |
| Insula (27) | -30.5 | 22.5 | -3.5 |
| Superior medial frontal gyrus ([SMFG], 28) | -0.5 | 50.5 | 29.5 |
| Inferior frontal gyrus ([IFG], 29) | -48.5 | 34.5 | -0.5 |
| Right inferior frontal gyrus ([R IFG], 30) | 53.5 | 22.5 | 13.5 |
| Middle frontal gyrus ([MiFG], 31) | -41.5 | 19.5 | 26.5 |
| Inferior parietal lobule ([IPL], 32) | -53.5 | -49.5 | 43.5 |
| Left inferior parietal lobue ([R IPL], 33) | 44.5 | -34.5 | 46.5 |
| Supplementary motor area ([SMA], 34) | -6.5 | 13.5 | 64.5 |
| Superior frontal gyrus ([SFG], 35) | -24.5 | 26.5 | 49.5 |
| Middle frontal gyrus ([MiFG], 36) | 30.5 | 41.5 | 28.5 |
| Hippocampus ([HiPP], 37) | 23.5 | -9.5 | -16.5 |
| Left inferior parietal lobule ([L IPL], 38) | -47.5 | 5.5 | 22.5 |
| Middle cingulate cortex ([MCC], 39) | -15.5 | 20.5 | 37.5 |
| Inferior frontal gyrus ([IFG], 40) | 39.5 | 44.5 | -0.5 |
| Middle frontal gyrus ([MiFG], 41) | -26.5 | 47.5 | 5.5 |
| Hippocampus ([HiPP], 42) | -24.5 | -36.5 | 1.5 |
| **Default-mode network (DM)** | | | |
| Precuneus (43) | -8.5 | -66.5 | 35.5 |
| Precuneus (44) | -12.5 | -54.5 | 14.5 |
| Anterior cingulate cortex ([ACC], 45) | -2.5 | 35.5 | 2.5 |
| Posterior cingulate cortex ([PCC], 46) | -5.5 | -28.5 | 26.5 |
| Anterior cingulate cortex ([ACC], 47) | -9.5 | 46.5 | -10.5 |
| Precuneus (48) | -0.5 | -48.5 | 49.5 |
| Posterior cingulate cortex ([PCC], 49) | -2.5 | 54.5 | 31.5 |
| **Cerebellar network (CB)** | | | |
| Cerebellum ([CB], 50) | -30.5 | -54.5 | -42.5 |
| Cerebellum ([CB], 51) | -32.5 | -79.5 | -37.5 |
| Cerebellum ([CB], 52) | 20.5 | -48.5 | -40.5 |
